# Integrating network annotation from multiple correlated traits to improve polygenic risk scores based on GWAS summary statistics

**DOI:** 10.21203/rs.3.rs-9073777/v1

**Published:** 2026-04-13

**Authors:** Lirong Zhu, Xuewei Cao, Shuanglin Zhang, Qiuying Sha

**Affiliations:** 1 Department of Bioinformatics, School of Basic Medical Sciences, Tianjin Medical University, Tianjin 300070, China.; 2 Department of Mathematical Sciences, Michigan Technological University, Houghton, Michigan, USA; 3 Center for Statistical Genetics, The Gertrude H. Sergievsky Center, Columbia University, New York, NY, USA, 10032; 4 Computational and Systems Biology, Sloan Kettering institute, Memorial Sloan Kettering Cancer Center, New York, NY, USA, 10065

**Keywords:** Network annotation, Polygenic risk score, Multiple correlated traits, GWAS summary statistics

## Abstract

Polygenic risk scores (PRS) are valuable tools for predicting disease risk based on genetic information, with potential impacts on disease prevention and early treatment strategies. Although thousands of disease-associated genetic variants have been identified through genome-wide association studies (GWAS), the accuracy of genetic risk prediction for most diseases remains moderate and challenging. In this paper, we introduce NetPRS, a novel method that utilizes a penalized regression model and leverages network annotation information to enhance PRS prediction. This network annotation is obtained from a genotype-phenotype bipartite network (GPN), where multiple SNPs and traits are linked based on association strengths obtained from GWAS summary statistics. The network annotation allows for the incorporation of information from relevant traits into the PRS prediction for the target trait. Compared to state-of-the-art risk prediction methods, NetPRS consistently achieves improved prediction accuracy in both simulation studies and real data analysis.

## Introduction

Polygenic risk scores (PRS) have emerged as valuable tools in predicting disease risk based on genetic information. They are calculated by summing the effects of multiple genetic variants across the genome, weighted by the effect sizes. PRS can provide insights into the genetic architecture of complex traits and diseases, aiding in personalized medicine and disease prevention.

PRS has been widely utilized across various fields. In disease risk prediction, PRS serves as an estimator of the genetic component, integrating with non-genetic risk factors to predict disease risk and trajectories [1]. In pleiotropic association detection, PRS-PheWAS identifies the association between PRS and multiple traits, overcoming the challenge of identifying pleiotropic variants with small effect sizes by leveraging a combination of genetic variants [2]. The accurate construction of PRS stands as a crucial component in advancing the understanding of complex traits and diseases. For the majority of common diseases and quantitative traits, PRS currently has a relatively low overall prediction accuracy across individuals in the general population. Importantly, PRS accuracy is expected to improve along with increasing the sample size of GWAS, the availability of new genomic information from omics studies, and the development of advanced PRS methods.

Many PRS methods have been developed to improve prediction accuracy[3]. Sparse-modeling approaches, such as the C+T model [3] and lassosum [4], select a subset of SNPs for PRS construction, whereas Bayesian frameworks estimate posterior effect sizes under different priors [5,6]. Although these methods are computationally efficient, recent studies have shown that conventional PRS generation may limit prediction performance and discard useful information [7]. To further improve PRS prediction, several methods incorporate additional information: multivariate PRS methods leverage genetically correlated traits [8, 9], and functional annotation-based methods utilize biological annotations of SNPs to refine weighting [10–12]. For example, LDpred-funct improves polygenic prediction by incorporating trait-specific functional priors [12]. Furthermore, leveraging pleiotropy—the shared genetic effects across multiple correlated traits—can enhance the estimation of SNP effect sizes for the target trait [11, 13–15]. Several multivariate PRS methods have been developed to leverage traits correlated or relevant to the target trait, further facilitating prediction accuracy [16–18].

Despite the effectiveness of incorporating additional information on the prediction of PRS, there are some limitations. Some methods make the simple assumption that all SNPs have non-zero effects for the target and relevant traits. This cannot capture the shared genetic and nongenetic architecture between the target and relevant traits [7]. Some methods use a pairwise modeling framework, incorporating one relevant trait at a time, and cannot accommodate more traits simultaneously [11]. Additionally, integrating more relevant traits into PRS increases the model complexity, making it more challenging to estimate SNP effect sizes.

Here, we propose NetPRS, a novel method that utilizes a penalized regression model and leverages network annotation information to enhance PRS prediction. This network annotation is obtained from a genotype-phenotype bipartite network (GPN), wherein multiple SNPs and multiple traits are linked based on association strengths obtained from GWAS summary statistics [19]. The bipartite structure of the GPN enables us to capture and characterize the intricate relationships between SNPs and traits, providing a framework that is both reproducible and accurately reveals biological interactions. Importantly, the network annotation allows for the incorporation of information from relevant traits into the PRS prediction of the target trait. The network annotation metric, representing either degree centrality or approximate betweenness centrality, serves as a crucial indicator, with SNPs exhibiting high network annotation being more likely to be pleiotropic variants. This approach enhances the accuracy and interpretability of PRS analysis by considering the broader genetic landscape and the complex relationships between genetic variants and phenotypes. NetPRS utilizes GWAS summary statistics and a population-matched linkage disequilibrium (LD) reference as input, which enhances its flexibility and accessibility. Additionally, leveraging GWAS summary statistics enables the fine-tuning of PRS models through methods such as Parameter-tuning Using Marginal Association Statistics (PUMAS) [20]. This approach allows NetPRS to refine its predictions when individual-level data are unavailable.

## Method

### Method overview

**Figure 1.**
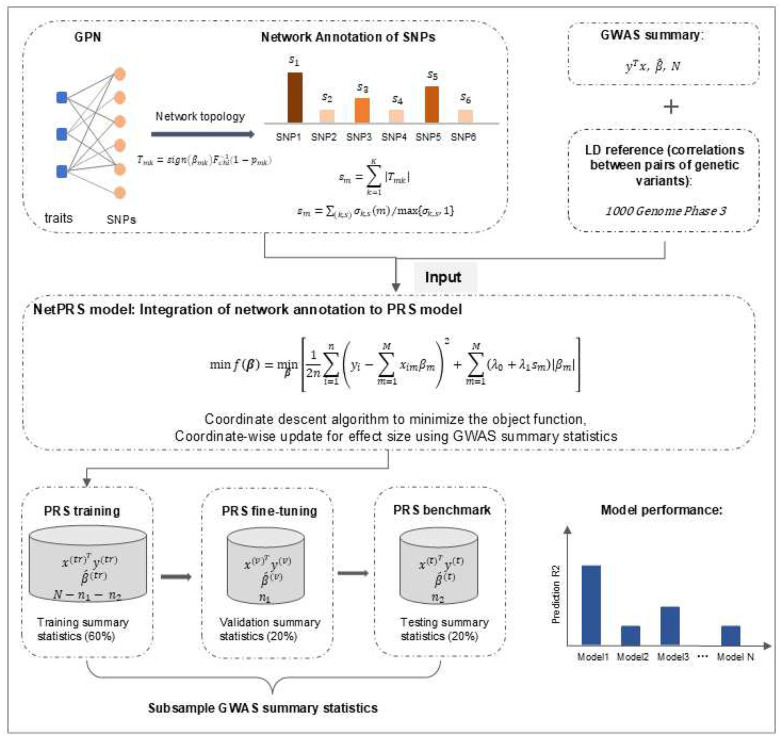
Overview of the NetPRS method. NetPRS is developed to improve the PRS construction by integrating SNP network annotation into the PRS model. NetPRS utilizes GWAS summary statistic, external LD reference from 1000 Genome Project, and SNP network annotations as the input (top). GWAS summary statistics from relevant traits are used to construct the bipartite genotype-phenotype network and subsequently extract the SNP annotations (top left). Then we construct the penalized regression model and use the coordinate descent algorithm to estimate the effect size (middle). Alternatively, when individual-level data are unavailable, we subsample the GWAS summary statistics for validation and testing to tune parameters and evaluate the model’s performance (bottom).

PRS is computed as the weighted sum of genotypes across SNPs, with the SNP effect size estimates used as the weights. To enhance genetic prediction of a target trait, NetPRS incorporates the shared genetic architecture with correlated traits, represented by network topology extracted from a GPN [21]. Degree centrality and approximate betweenness centrality of a SNP serve as indicators of pleiotropic effects. Effect sizes are estimated via a penalized regression model incorporating these network annotations based on GWAS summary statistics. Model performance is evaluated by splitting the data into training, validation, and testing sets without requiring individual-level data [20]. This strategy is particularly beneficial in cases where individual-level data is not available. The NetPRS workflow is illustrated in Figure 1.

### Quantifying network annotation based on GPN

Network annotations are obtained from a GPN in two steps.

*Step 1*: *Obtain the relevant traits correlated to the target trait using cross-trait LD Score regression[*22*]*. The cross-trait LD score regression is defined as

$$E\left[z_{1j}z_{2j}|l_{j}\right]=\frac{\sqrt{N_{1}N_{2}}\rho_{g}}{M}l_{j}+\frac{\rho N_{s}}{\sqrt{N_{1}N_{2}}}$$

where $z_{ij}$ is the z-score for trait i and SNP $j,N_{i}$ is the sample size of trait i,M is the number of SNPs, $\rho_{g}$ is the genetic covariance, ρ is the phenotypic correlation among the $N_{s}$ overlapped samples, and $l_{j}$ is the LD score of the variant j [23]. We can estimate the genetic covariance $\rho_{g}$ by regressing $z_{1j}z_{2j}$ against $\frac{\sqrt{N_{1}N_{2}}l_{j}}{M}$ based on the above regression model. The trait serves as a relevant trait if it is genetically correlated with the target trait.

*Step 2: Obtain the network annotation from GPN*. We consider a target trait and K relevant traits, assuming all traits share the same M SNPs for simplicity. We use the GWAS summary statistics of K relevant traits and M SNPs to construct a signed GPN. Let $\text{T}=\left(T_{mk}\right)$ represent an M×K adjacency matrix for the signed bipartite network, where $T_{mk}=\text{sign}\left({\overset{ˆ}{\beta }}_{mk}\right)F_{chi}^{-1}\left(1-p_{mk}\right)$ denoting the association strength between the $m^{th}$ SNP and the $k^{\text{th}}$ trait; ${\overset{ˆ}{\beta }}_{mk}$ and $p_{mk}$ represent the effect size of the $m^{\text{th}}$ SNP on the $k^{\text{th}}$ trait and the corresponding p-value obtained from the GWAS summary statistics based on the marginal association test. $\text{sign}\left({\overset{ˆ}{\beta }}_{mk}\right)=1$ if ${\overset{ˆ}{\beta }}_{mk}\geq 0$ and $\text{sign}\left({\overset{ˆ}{\beta }}_{mk}\right)=-1$ if ${\overset{ˆ}{\beta }}_{mk}<0$. We can derive two network annotations from the bipartite network: degree centrality and approximate betweenness centrality. For the $m^{\text{th}}$ SNP, the weighted degree centrality is defined as $d_{m}=\sum_{k=1}^{K}\left|T_{mk}\right|$. The approximate betweenness centrality is defined as $b_{m}=\sum_{\left(k,s\right)}\sigma_{k,s}(m)/\text{max}\left(\sigma_{k,s},1\right)$, where $\sigma_{k,s}(m)$ is the number of shortest paths between the $k^{th}$ and $s^{\text{th}}$ trait that pass through the $m^{\text{th}}$ SNP. The SNP degree centrality and approximate betweenness centrality are calculated using a well-defined GPN introduced in the reference study [19, 21].

These network annotations measure the importance of SNPs among the relevant traits. The SNP with a high degree centrality across traits is more likely to be pleiotropic, owing to its strong connections with multiple traits. A SNP with high approximate betweenness can be considered an important connector between multiple traits. The network annotation captures the shared genetic architecture among traits, providing valuable information for understanding the underlying genetic mechanisms. Integrating this network annotation into the model can effectively leverage the pleiotropic effects of genetic variants across multiple traits, thereby enhancing the prediction accuracy for the target trait.

### Incorporating network annotation in the penalized regression model

Let $\text{Y}={\left(y_{1},\ldots ,y_{n}\right)}^{T}$ represent the target trait and let $\text{X}=\left(x_{ij}\right)$ be a n×M genotype matrix for n subjects and M SNPs. Assuming that the target trait and the genotype for each SNP is normalized to have 0 mean and unit variance. We construct a penalized regression model by including a penalty term based on the network annotation, and then derive the regularized estimates of effect size β by minimizing the object function:

$$\text{min}\;f(\beta )=\underset{\beta }{\text{min}}\left[\frac{1}{2n}\sum_{i=1}^{n}{\left(y_{i}-\sum_{m=1}^{M}x_{im}\beta_{m}\right)}^{2}+\sum_{m=1}^{M}\left(\lambda_{0}+\frac{\lambda_{1}}{s_{m}}\right)\left|\beta_{m}\right|\right]$$

where $\lambda_{0}$ and $\lambda_{1}$ are tuning parameters, $s_{m}$ is the network annotation (degree centrality or approximate betweenness centrality) for the $m^{\text{th}}$ SNP, and $\beta =\left(\beta_{1},\ldots ,\beta_{M}\right)$ represents the effect size vector of the target trait for M SNPs. We use the coordinate descent method to minimize the object function[24]. Assuming that we have the estimation $\tilde{\beta }=\left({\overset{ˆ}{\beta }}_{l}^{\left(t\right)}\right)$ for l≠m,l∈1,…,M at the $t^{th}$ iteration, we then partially optimize the object function with respect to $\beta_{m}$. If $\beta_{m}>0$, then

$${\left.\frac{\partial f}{\partial \beta_{m}}\right|}_{\beta =\tilde{\beta }}=-\frac{1}{n}\sum_{i=1}^{n}\left(y_{i}-\sum_{l\neq m}x_{il}{\overset{ˆ}{\beta }}_{l}^{\left(t\right)}\right)x_{im}+(\lambda_{0}+\lambda_{1}\cdot \frac{1}{s_{m}})$$

A similar expression exists if $\beta_{m}<0$. Then the coordinate-wise update of $\beta_{m}$ at the $(t+1{)}^{th}$ iteration is ${\overset{ˆ}{\beta }}_{m}^{\left(t+1\right)}\leftarrow S\left(\frac{1}{n}\sum_{i=1}^{n}\left(y_{i}-\sum_{l\neq m}x_{il}{\overset{ˆ}{\beta }}_{l}^{\left(t\right)}\right)x_{im},\lambda_{0}+\frac{\lambda_{1}}{s_{m}}\right)$. Denote $w_{m}^{\left(t\right)}=\frac{1}{n}\sum_{i=1}^{n}\left(y_{i}-\sum_{l\neq m}x_{il}{\overset{ˆ}{\beta }}_{l}^{\left(t\right)}\right)x_{im}$ and $\tau_{m}=\lambda_{0}+\frac{\lambda_{1}}{s_{m}}$, the soft thresholding operator $S\left(w_{m}^{\left(t\right)},\tau_{m}\right)$ is defined as

$${\overset{ˆ}{\beta }}_{m}^{\left(t+1\right)}=S\left(w_{m}^{\left(t\right)},\tau_{m}\right)=\left\{\begin{array}{ll} 0 & \text{if}\left|w_{m}^{\left(t\right)}\right|\leq \tau_{m}\\ \text{sign}\left(w_{m}^{\left(t\right)}\right)\left||w_{m}^{\left(t\right)}|-\tau_{m}\right|, & \text{if}\left|w_{m}^{\left(t\right)}\right|>\tau_{m} \end{array}\right.$$

where $\lambda_{0}$ is the baseline penalty applying to all SNPs, $\lambda_{1}$ is an SNP-specific penalty related to network annotation, and $\lambda_{0},\lambda_{1}>0$. Intuitively, a SNP with a larger network annotation score (i.e., stronger pleiotropic connections across traits) receives a smaller penalty. We repeat this procedure until convergence is achieved.

### A network annotation-dependent model for PRS utilizing GWAS summary statistics

With the above model, our goal is to obtain the estimation of effect size β using GWAS summary statistics. Here, we update the beta estimates using a coordinate descent algorithm based on two terms $w_{m}^{\left(t\right)}$ and $\tau_{m}$ for SNP m in the $(t+1{)}^{\text{th}}$ iteration. For the first term, $w_{m}^{\left(t\right)}=\frac{1}{n}\sum_{i=1}^{n}\left(y_{i}-\sum_{l\neq m}x_{il}{\overset{ˆ}{\beta }}_{l}^{\left(t\right)}\right)x_{im}=\frac{1}{n}\sum_{i=1}^{n}y_{i}x_{im}-\frac{1}{n}\sum_{i=1}^{n}\sum_{l\neq m}x_{il}x_{im}{\overset{ˆ}{\beta }}_{l}^{\left(t\right)}$, we have $\frac{1}{n}\sum_{i=1}^{n}y_{i}x_{im}={\tilde{\beta }}_{m}$, where ${\tilde{\beta }}_{m}$ represents the marginal effect of SNP m after standardizing the genotype for each SNP and can be obtained directly from GWAS summary statistics. Let ${\overset{ˆ}{\rho }}_{lm}=\frac{1}{n}\sum_{i=1}^{n}x_{il}x_{im}$, which is the empirical genotype correlation between SNP l and SNP m. Ideally, ${\overset{ˆ}{\rho }}_{lm}$ can be calculated based on the genotype data from the training dataset. However, we also can approximate ${\overset{ˆ}{\rho }}_{lm}$ using the genotype from the reference data with the same ancestry, such as 1000 Genome Project [25]. The second term, $\tau_{m}=\lambda_{0}+\frac{\lambda_{1}}{s_{m}}$, is dependent on the network annotation, which is obtained from the bipartite genotype-trait network based on GWAS summary statistics. Using the above model, we can obtain the estimation of effect size $\hat{\beta }=\left({\overset{ˆ}{\beta }}_{m}\right)$ for m∈1,2,…,M. Then the PRS of a target trait is calculated as $PRS={\text{X}}^{T}\hat{\beta }$.

### Model tuning and evaluation using GWAS summary statistics

We split the samples into training, validation and testing datasets when individual-level data are available. These three datasets are used for training the model, selecting tuning parameters, and evaluating model performance, respectively. We develop and evaluate the proposed method in the context of a quantitative target trait. Its performance is assessed via the coefficient of determination (R^2^), which quantifies the proportion of genetic variation in the target trait predicted by the model.

We use the PUMAS method to select the optimal tuning parameter and evaluate the model performance if the individual-level data is unavailable [20]. This involves subsampling the GWAS summary statistics into training and validation sets, with the latter used for tuning parameter selection. Specifically, the statistic ${\text{X}}^{T}\text{y}$ can be calculated from the full summary statistics and the full sample size N is known. We can partition ${\text{X}}^{T}\text{y}$ into training set ${\text{X}}^{(tr)T}{\text{y}}^{\left(\text{tr}\right)}$ and validation set ${\text{X}}^{(v)T}{\text{y}}^{\left(v\right)}$. The statistic ${\text{X}}^{(tr)T}{\text{y}}^{\left(\text{tr}\right)}$ related to the training set can be generated by the distribution ${\text{X}}^{(tr)T}{\text{y}}^{\left(\text{tr}\right)}\left|{\text{X}}^{T}\text{y}\sim \text{N}\left(\frac{N-n}{N}{\text{X}}^{T}\text{y},\frac{N-n}{N}𝚺\right)\right.$, where n is the predefined sample size of training dataset, 𝚺 is the observed covariance matrix of ${\text{X}}^{T}\text{y}$ which can be estimated using GWAS summary statistics or a reference panel such as 1000 Genome Project [25]. After simulating the term ${\text{X}}^{(tr)T}{\text{y}}^{\left(\text{tr}\right)}$, we can obtain the validation set by ${\text{X}}^{(v)T}{\text{y}}^{\left(v\right)}={\text{X}}^{T}\text{y}-{\text{X}}^{(tr)T}{\text{y}}^{\left(\text{tr}\right)}$. The testing dataset is generated following an identical procedure, simply substituting the validation partition with a testing partition in the formula above.

Subsequently, we evaluate the model performance using the coefficient of determination $\left(R^{2}\right)$, the average $R^{2}$ across 10 random splits using GWAS summary statistics [20]. The $R^{2}$ on the validation or testing dataset can be approximated as

$$R^{2}=\frac{{\left(\sum_{i=1}^{n}y_{i}^{\left(v\right)}{\overset{ˆ}{y}}_{i}^{\left(v\right)}-n{\overset{‾}{y}}^{\left(v\right)}{\bar{\overset{ˆ}{y}}}^{\left(v\right)}\right)}^{2}}{\sum_{i=1}^{n}{\left(y_{i}^{\left(v\right)}-{\overset{‾}{y}}^{\left(v\right)}\right)}^{2}\sum_{i=1}^{n}{\left({\overset{ˆ}{y}}_{i}^{\left(v\right)}-{\bar{\overset{ˆ}{y}}}^{\left(v\right)}\right)}^{2}}\approx \frac{{\left(\frac{1}{n}\sum_{m=1}^{M}w_{m}{\text{X}}_{\text{m}}^{(v)T}{\text{y}}^{\left(\text{v}\right)}\right)}^{2}}{N\;{max}_{m}\left[SE{\left({\overset{ˆ}{\beta }}_{m}\right)}^{2}{\overset{ˆ}{\sigma }}_{m}^{2}\right]\sum_{m=1}^{M}w_{m}^{2}{\overset{ˆ}{\sigma }}_{m}^{2}}$$

where $w_{m}$ is the estimation of effect size using our method and compared methods, ${\overset{ˆ}{\sigma }}_{m}^{2}$ is a minor allele frequency (MAF) based estimator of $E\left(X_{m}^{2}\right)$ for the $m^{\text{th}}$ SNP which can be estimated from the reference panel. All in all, the above approach allows us to benchmark the predictive accuracy of various methods, providing insights into their effectiveness in predicting the target trait.

### Compared methods

We compare NetPRS against four methods: C+T [26], lassosum [4], LDpred-inf [5], and PANPRS [27]. The C+T method selects independent SNPs via LD-pruning and p-value thresholding [26]. The lassosum method estimates effect sizes through Lasso regression on GWAS summary statistics and a reference panel [4]. The LDpred-inf method approximates posterior mean effects under an infinitesimal model accounting for LD [5]. The PANPRS method models pleiotropy across correlated phenotypes [27]. The first three are univariate methods, while PANPRS is a multi-trait method. For multi-trait methods, traits with significant genetic correlation (p<0.05/K) with the target trait, identified by cross-trait LD score regression [22], are used as relevant traits.

### Simulations

In our study, we evaluate PRS prediction of a target trait by incorporating network annotation based on relevant traits. The simulation involves three steps:

**Step 1:** Multiple quantitative traits are simulated using the PheGen simulator [28], which models shared genetic and environmental architecture under a matrix normal distribution Y=XB+E, where Y is a $N_{s}\times K$ matrix for $N_{s}$ overlapped individuals and K traits where the first trait is defined as the target trait. X is a $N_{s}\times M$ genotype matrix which is standardized to have a mean of 0 and a variance of 1, matrix B denotes the effect of the genetic component with dimension M×K, and E denotes non-genetic effects with dimension $N_{s}\times K$. We can model B and E as:

$$\text{B}\sim {𝓜𝓝}_{M\times K}(\text{O},\text{I},\frac{1}{M\cdot p}\left[\begin{array}{cccc} h_{1}^{2} & \rho_{g_{12}} & \ldots & \rho_{g_{1K}}\\ \rho_{g_{12}} & h_{2}^{2} & \cdots & \rho_{g_{2K}}\\ \ldots & \ldots & \ldots & \ldots \\ \rho_{g_{1K}} & \rho_{g_{2K}} & \cdots & h_{K}^{2} \end{array}\right])$$

$$\text{E}\sim {𝓜𝓝}_{N_{S}\times K}(\text{O},\text{I},\left[\begin{array}{cccc} 1-h_{1}^{2} & \rho_{e_{12}} & \cdots & \rho_{e_{1K}}\\ \rho_{e_{12}} & 1-h_{2}^{2} & \cdots & \rho_{e_{2K}}\\ \cdots & \ldots & \cdots & \cdots \\ \rho_{e_{1K}} & \rho_{e_{2K}} & \cdots & 1-h_{K}^{2} \end{array}\right])$$

where I is the M×M identity matrix, O is an M×K matrix with each element set to be 0, $\rho_{g_{ij}}$ and $\rho_{e_{ij}}$ are the genetic and non-genetic covariance between trait i and trait j on the overlapped individuals, respectively, and $h_{k}^{2}$ is the heritability explained by the variants in a region for the $k^{\text{th}}$ trait and it is equally distributed among all SNPs in this region. We use the same genotype data for the overlapped samples and independently simulated genotype data for the non-overlapped individuals. The genotype is generated by the calibration coalescent model (COSI) [29]. We simulate the SNP effect sizes and non-genetic effect sizes under the polygenic architecture described in the above model. Then the simulated traits are defined as the summation of genetic and non-genetic effects.

**Step 2:** We use the Plink tool [30] to obtain the GWAS summary statistics based on the simple linear regression model. Subsequently, we employ cross-trait LD score regression to select relevant traits correlated with the target trait. This ensures that the genetic correlation between the target trait and each relevant trait is significant, as determined by the Idsc command line tool [22]. Next, we construct a GPN based on GWAS summary statistics using GPN package [19], from which degree centrality and approximate betweenness centrality are extracted as SNP network annotations.

**Step 3:** Network annotations are integrated into the penalized regression. When individual data are available, we randomly split all samples into training, validation, and testing datasets with the proportions of 60%, 20%, and 20%, respectively. In cases where individual data are not available, we use the PUMAS method to subsample the GWAS summary statistics into training, validation, and testing datasets, maintaining the same proportions as mentioned above. Performance is averaged over ten replicates.

Simulation settings vary the number of traits (K=5 or 10), heritability ($h^{2}=0.2$ or 0.6), genetic correlation ($\rho_{g}=0.6$ or 0.1), and sample overlap scheme (non-overlapped, partially overlapped with 2,000 shared individuals, or completely overlapped). All traits have equal sample size N=5,000,M=5,000 SNPs, and a causal variant proportion p=0.01. Environmental correlation is fixed at $\rho_{e}=0.1$. Overall, there are 24 simulation settings and all simulation settings are listed in Table S1.

### Real data analysis

Chronic obstructive pulmonary disease (COPD) is a significant public health concern worldwide due to its high prevalence, morbidity, and mortality [31–34]. The Genetic Epidemiology of COPD Study (COPDGene) is a large multicenter study of the genetic and environmental factors contributing to COPD [32]. We assess the model prediction of the proposed method NetPRS with comparison methods (PANPRS, C+T, lassosum, and LDpred-inf) using real data from the COPDGene study, focusing on the non-Hispanic white (NHW) population (https://www.copdgene.org/). Our analysis includes nine key COPD-related traits: Body Mass Index (BMI), Pack years (ATS), Percent gas trap, Number of COPD exacerbations, Percent emphysema, Airway Wall Area (Pi10), Upper Third/Lower Third ratio CT Slicer, Distance walked in six minutes, and FEV1/FVC (post-bronchodilator) [35–42]. GWAS quality control (QC) criteria included: genotyping rate > 0.99, sample missingness < 0.02, Hardy-Weinberg Equilibrium (HWE) $P>1\times 10^{-6}$, minor allele frequency (MAF) > 1% and imputation ‘info score’ > 0.8 [3]. A total of 5,430 individuals and 630,860 SNPs from autosomes are included in the real data analysis after quality control. To demonstrate the applicability of NetPRS in scenarios where individual-level data are unavailable, we also apply the method using only GWAS summary statistics. We first perform the marginal association test to obtain the GWAS summary statistics for these 9 COPD-related traits and utilize the external LD reference panel from the 1000 Genome Project European samples as input, eliminating the need for individual-level data. We retain 497,497 SNPs that intersect with the 1000 Genomes Phase III data of European ancestry for our study. To obtain the SNP network annotation, we select one of the nine traits as the target trait and use the remaining traits as relevant traits. Then, we construct the GPN with all relevant traits to extract SNP network annotations.

To assess the prediction performance of PRS using both individual-level data and GWAS summary statistics, we utilize data from the COPDGene. For samples with individual-level data, we divide the dataset into training, validation, and testing subsets. For samples lacking individual-level data, we partition the GWAS summary statistics into corresponding training, validation, and testing subsets.

## Results

### Simulation results

**Figure 2.**
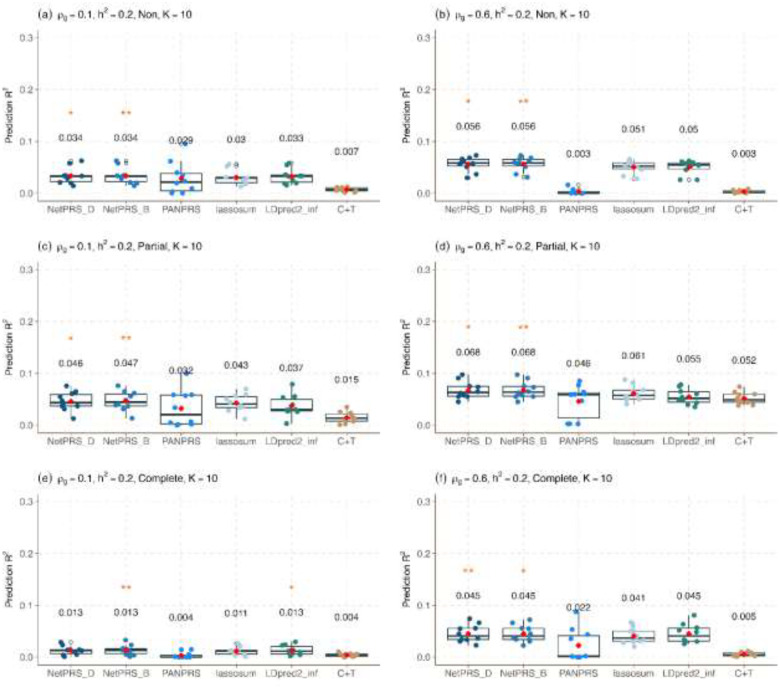
Predictive performance of NetPRS and other methods in simulations under different genetic correlations. The boxplot illustrates the prediction $R^{2}$ across ten simulation replicates for each method in six simulation settings. The average value of $R^{2}$ across ten replicates is shown above the boxplot. The first two are based on NetPRS but employ different network annotations. The last four are comparison methods including PANPRS, lassosum, LDpred-inf, and C+T. Results are presented with a heritability of 0.2. The methods with the best performance are identified with two asterisks, while the second-best PRS method is denoted with a single asterisk. The simulation settings (a)-(f) feature different genetic correlations (0.1 or 0.6) under non-overlapping (a) and (b), partially overlapping (c) and (d), and completely overlapping (e) and (f).

We evaluate the prediction accuracy of NetPRS using two network annotations—NetPRS_D (degree centrality) and NetPRS_B (approximate betweenness centrality)—against PANPRS, lassosum, LDpred-inf, and C+T. In our simulations, we vary the number of traits (K=5 or K=10), SNP heritability ($h_{k}^{2}=0.2$ or $h_{k}^{2}=0.6$), and genetic correlation ($\rho_{g}=0.1$ or $\rho_{g}=0.6$), and employed three schemes for the overlapped sample studies (no overlap, partial overlap, complete overlap), resulting in 24 simulation scenarios. Based on the simulation results (Figures 2, Figures S1–S8), NetPRS consistently ranks as the most accurate method in 18 out of 24 simulation settings and second-most accurate in 5 out of the remaining 6, achieving an accuracy gain of 8.4% over the second-best comparison method in those 18 settings. NetPRS particularly excels under high heritability, strong genetic correlation, and a large number of traits.

NetPRS leverages shared genetic architecture between target and relevant traits, with stronger genetic correlation further improving accuracy (Figure 2). With a heritability of $h^{2}=0.2$ and K=10 traits, NetPRS outperforms other methods, and achieves accuracy gains of 2.9% and 8.9% over the second-best comparison method in non-overlapped settings as the genetic correlation $\rho_{g}$ increases from 0.1 to 0.6. Similarly, in partially-overlapped settings, NetPRS achieves an average accuracy gain of 8.5% and 10% over the second-best method as $\rho_{g}$ increases from 0.1 to 0.6. In complete-overlapped settings, sample overlap attenuates the advantage of multi-trait methods, and NetPRS performs comparably to the best univariate method, consistent with the observation that complete overlap reduces performance for multi-trait approaches. Results for other heritability and trait-number combinations show consistent patterns (Figure S1–S3).

The simulation results provide deeper insights into method performance under different genetic architectures. As heritability $h^{2}$ increases from 0.2 to 0.6, prediction performance improves approximately 2- to 3-fold for most methods. While sample overlap does not affect univariate PRS methods, it reduces performance for multi-trait approaches in completely overlapped settings (Figure S4–S7). Moreover, incorporating more relevant traits consistently enhances the performance of multi-trait PRS methods. For example, in a non-overlapping scenario with fixed genetic correlation $\rho_{g}=0.1$ and heritability $h^{2}=0.2$, prediction $R^{2}$ typically improves around 1- to 2-fold for multiple-traits PRS methods (Figure S8).

The prediction performance of NetPRS can be evaluated regardless of individual data availability. In settings where individual-level data are available, we split the samples into training, validation, and test sets. When only GWAS summary statistics are available, $R^{2}$ can be estimated using an external reference panel, and the summary statistics are subsampled to form training, validation, and test subsets. To validate that both approaches yield consistent results, we conducted simulations comparing $R^{2}$ estimates derived from individual data with those from summary statistics. The results show that the Pearson correlation of average $R^{2}$ is greater than 0.719 for all methods except for C+T (Figure S9). The lower concordance for C+T likely reflects its threshold-based SNP selection, which is sensitive to the particular sample used and therefore less stable across individual-data and summary-statistic modes than regression-based methods.

### PRS prediction accuracy for COPD related traits

**Figure 3.**
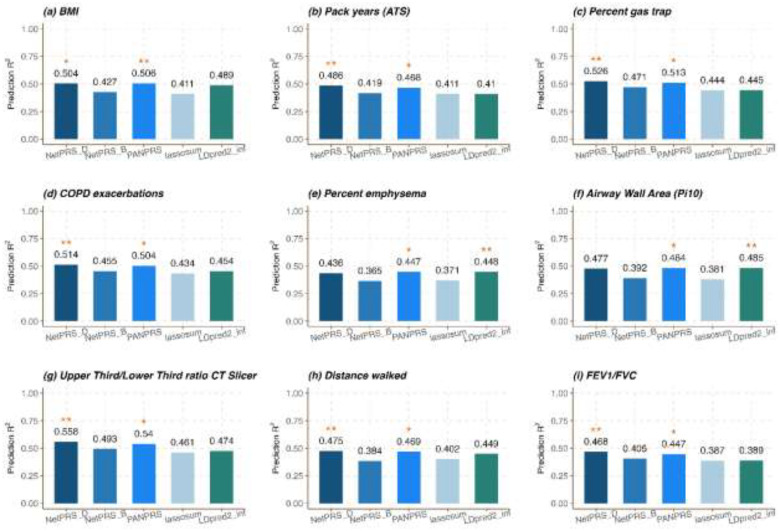
PRS prediction accuracy for COPD related traits based on individual-level data. We randomly split the sample into a training set of 60% individuals, a validation set of 20% individuals, and a test set of 20% individuals. The Bar plot displays the average $R^{2}$ across ten replicates for different methods. The methods with the best performance are identified with two asterisks, while the second-best PRS method is denoted with a single asterisk.

We assess the performance of NetPRS and comparison methods in COPD-related traits, focusing on the 9 traits in the COPDGene study. Our analysis involves 5,430 individuals and 497,497 SNPs. For multiple trait PRS methods, we designate each trait as the target trait and select relevant traits from the remaining 8 traits for analysis. We first fit the PRS models using the training set, fine-tune the parameters using the validation set, and then assess model performance in the test set for each trait, provided individual-level data is available. The results are summarized in Figure 3. Overall, NetPRS outperforms other comparison methods in 6 out of 9 traits. Additionally, NetPRS ranks as the second-best method in 1 out of the remaining 3 traits. For the traits where NetPRS performs best, it demonstrates an average accuracy gain of 2.94% compared to the second-best method (Figure 3).

**Figure 4.**
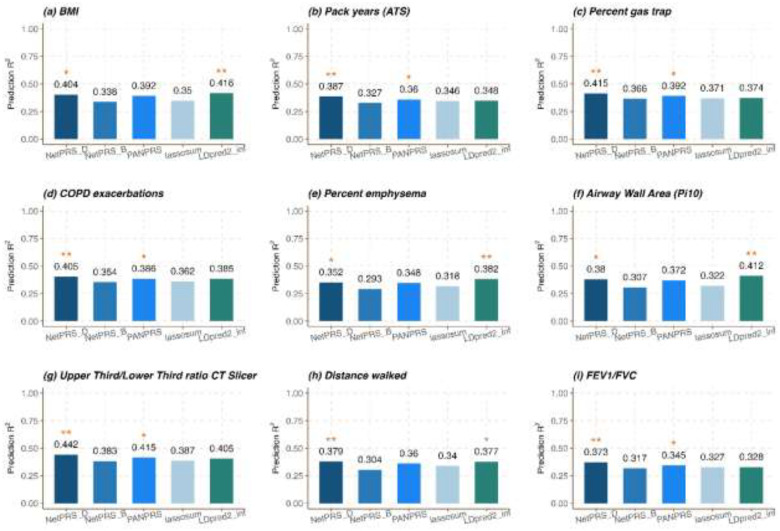
PRS prediction accuracy for COPD related traits based on GWAS summary statistics. We subsample GWAS summary statistics into 60% training, 20% validation, and 20% testing summary statistics. The LD reference from 1000 Genome project is used as the external input. The Bar plot displays the average $R^{2}$ across ten replicates for different methods. The method with the best performance is identified with two asterisks, while the second-best PRS method is denoted with a single asterisk.

To compare the performance of PRS methods based on individual-level data and GWAS summary statistics, we use the same 9 COPD-related traits from the COPDGene. Firstly, we perform the marginal association test to obtain the GWAS summary statistics. Then we subsample GWAS summary statistics into 60% training, 20% validation, and 20% testing summary statistics. The training summary statistics, along with an external LD reference from the 1000 Genomes Project, are used as input to train PRS models. Subsequently, we utilize the validation summary statistics to tune the parameters and evaluate the model performance. Overall, NetPRS outperforms other methods in 6 out of 9 traits and ranks as the second-best method in the remaining 3 traits (Figure 4). NetPRS achieves an average accuracy gain of 5.57% compared to the second-best comparison method. The performance of PRS predictions based on individual-level and GWAS summary statistics is consistent across traits.

## Discussion

NetPRS provides a robust method for PRS prediction by integrating information from multiple relevant traits through a GPN and penalized regression with network annotation. The method achieves robust prediction performance across diverse genetic architectures and study designs, accommodating variations in the number of relevant traits, degrees of sample overlap, and genetic correlations between the target trait and relevant traits. By relying solely on GWAS summary statistics and a population-matched LD reference, such as data from the 1000 Genomes Project, NetPRS offers a flexible and accessible framework for PRS construction. Simulations and applications to the COPDGene Study demonstrate the method’s ability to enhance prediction accuracy by leveraging shared genetic information.

The NetPRS approach employs a GPN to model the shared genetic architecture between the target trait and relevant traits. By identifying pleiotropic SNPs--those influencing multiple traits--within the bipartite network, NetPRS assigns lower penalty to these SNPs in the penalized regression model, reflecting their greater pleiotropic relevance as indicated by high node centrality within the GPN. Network annotation further enhances prediction by incorporating information from relevant traits, enabling the method to capture complex SNP-trait relationships. NetPRS uses cross-trait LD score regression to select relevant traits, ensuring that only genetically correlated traits contribute to the model. This approach ensures that only genetically relevant traits contribute to the model, avoiding the inclusion of noise from unrelated phenotypes and thereby improving prediction performance. The performance of NetPRS correlates with the number of relevant traits and the strength of their genetic correlation with the target trait, with a greater number of traits and stronger correlations yielding more accurate predictions. In both simulation and real data analyses, degree centrality-weighted and betweenness centrality–weighted PRS consistently outperformed conventional approaches, with trait-dependent differences in their relative effectiveness (detailed in Figures S1–S3), concordant with our prior observation that both annotations are significantly enriched for disease heritability yet differ in enrichment magnitude across traits [19]. This discrepancy may arise because degree centrality measures the number of phenotypes a variant is associated with in the bipartite GPN, thereby prioritizing variants with broad pleiotropic effects, whereas betweenness centrality captures variants that lie on the shortest paths connecting distinct phenotype communities, emphasizing inter-module mediating roles that are not reflected by direct connectivity alone. These complementary topological perspectives support retaining both centrality measures to accommodate the heterogeneous genetic architectures underlying complex traits.

The accuracy of NetPRS depends on the careful selection of genetically correlated traits via cross-trait LD score regression, and requires a population-matched LD reference when individual-level data are unavailable. The flexible framework, which places no limit on the number of relevant traits, ensures scalability across diverse study designs. Future work may explore machine learning-based trait selection, refined genetic correlation estimation, integration of functional annotations or multi-omics data to further improve SNP prioritization, and extension to family-based GWASs as seen in animal breeding programs.

In conclusion, NetPRS advances PRS prediction by integrating multiple relevant traits through a GPN. Its ability to model shared genetic architecture, combined with its reliance on accessible GWAS summary statistics, establishes it as a valuable tool for genetic research. By exploring refined trait selection methods and incorporating additional biological data, NetPRS holds potential to further enhance the accuracy and applicability of genetic risk assessments.

## Supplementary Material

1

## Data Availability

The COPDGene dataset supporting the findings of this study is available through the dbGaP study page for COPDGene: https://www.ncbi.nlm.nih.gov/projects/gap/cgi-bin/study.cgi?study id=phs000179.v3.p2. From this page, users can navigate to the Authorized Access section, which links to dbGaP’s controlled-access application system for requesting the data. The relevant accession numbers are phs000179/HMB and phs000179/DS-CS-RD.

## Code availability

PLINK version 1.9 is available for download from https://www.cog-genomics.org/plink/1.9/ [30]. LDSC, a command-line tool for estimating heritability and genetic correlation from GWAS summary statistics, is accessible at https://github.com/bulik/ldsc [22]. Simulation data were generated using the PheGen R package, available for download from https://github.com/Julia-lirong/PheGen. Network topology calculations were performed using the GPN R package, which can be downloaded from https://github.com/Julia-lirong/GPN.
