# Supplementary material for: Integrating network annotation from multiple correlated traits to improve polygenic risk scores based on GWAS summary statistics
Source: Res Sq. 2026 Apr 13:rs.3.rs-9073777. Preprint. [Version 1] doi: 10.21203/rs.3.rs-9073777/v1 (PMC13105137; doi:10.21203/rs.3.rs-9073777/v1)

Supplementary

Table S1: The simulation settings involved varying the number of traits, the number of overlapping sample sizes, the genetic correlation, and SNP heritability.

	Ns	K	M	ρ_g	ρ_e	h^2
Completely- overlapping	5000	5/10	5000	0.1/0.6	0.1	0.2/0.6
Non-overlapping	0					
Partially- overlapping	2000					

Note: Ns is the overlapping sample size among all traits. K is the number of traits. M is the total number of SNPs. ρ_g is the genetic correlation among the shared individuals. ρ_e is the non-genetic correlation among the shared individuals. h^2 is the heritability for these traits.

Figure S1. Predictive performances of NetPRS and other methods in simulations under different genetic correlations. The boxplot illustrates the prediction R^2 across ten simulation replicates for different methods in six simulation settings. The average value of R^2 across ten replicates is shown above the boxplot. The first two are based on NetPRS but employ different network annotations. The last four are compared methods including PANPRS, lassosum, LDpred-inf, and C+T. Results are presented with a heritability of 0.2 and 5 relevant traits. The methods with the best performance are identified with two asterisks, while the second-best PRS method is denoted with a single asterisk. The simulation settings (a)-(f) feature different genetic correlations (0.1 or 0.6) under non-overlapping (a) and (b), partially overlapping (c) and (d), and completely overlapping (e) and (f).

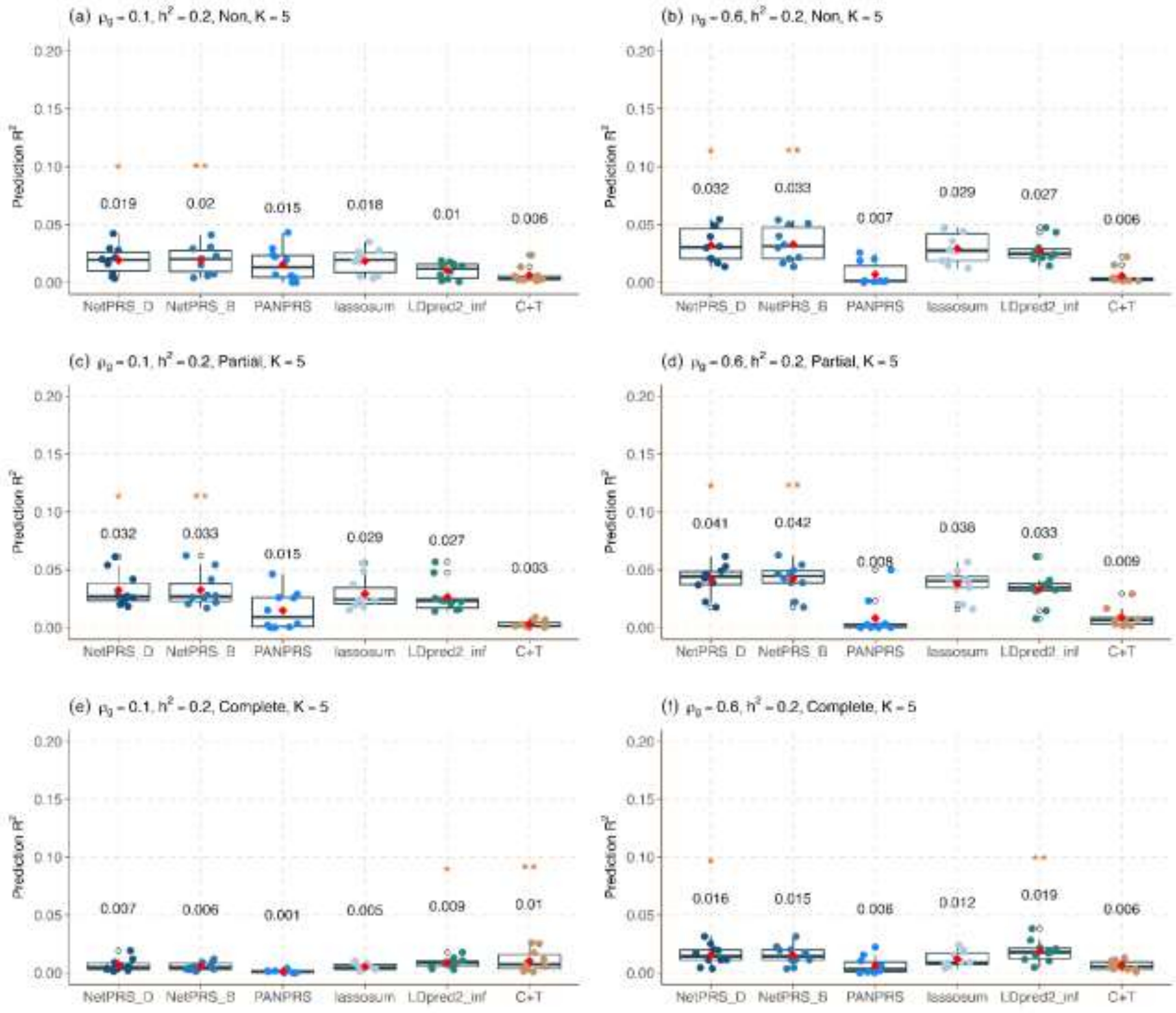

Figure S2. Predictive performances of NetPRS and other methods in simulations under different genetic correlations. The boxplot illustrates the prediction R^2 across ten simulation replicates for different methods in six simulation settings. The average value of R^2 across ten replicates is shown above the boxplot. The first two are based on NetPRS but employ different network annotations. The last four are compared methods including PANPRS, lassosum, LDpred-inf, and C+T. Results are presented with a heritability of 0.6 and 5 relevant traits. The methods with the best performance are identified with two asterisks, while the second-best PRS method is denoted with a single asterisk. The simulation settings (a)-(f) feature different genetic correlations (0.1 or 0.6) under non-overlapping (a) and (b), partially overlapping (c) and (d), and completely overlapping (e) and (f).

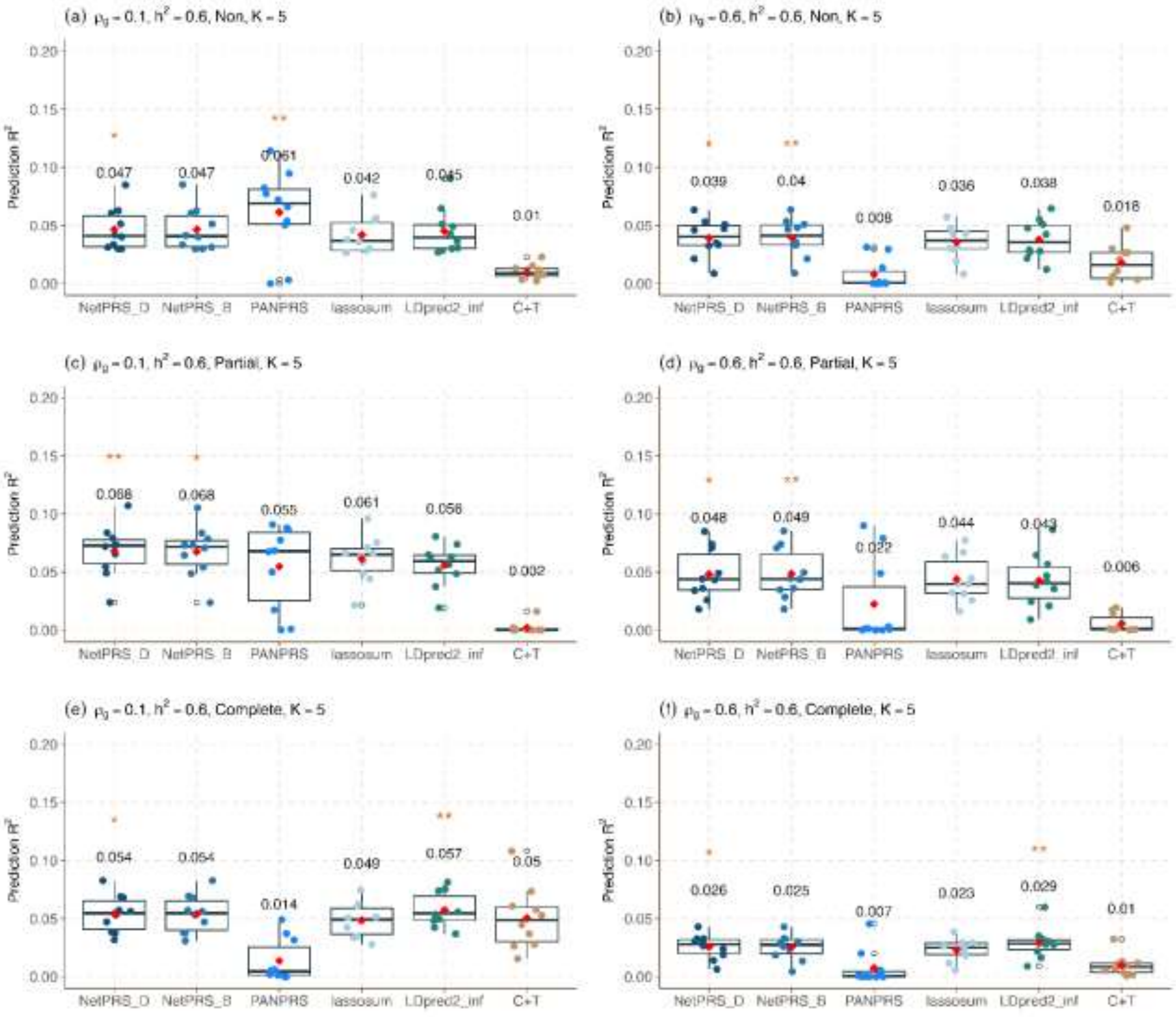

Figure S3. Predictive performances of NetPRS and other methods in simulations under different genetic correlations. The boxplot illustrates the prediction R^2 across ten simulation replicates for different methods in six simulation settings. The average value of R^2 across ten replicates is shown above the boxplot. The first two are based on NetPRS but employ different network annotations. The last four are compared methods including PANPRS, lassosum, LDpred-inf, and C+T. Results are presented with a heritability of 0.6 and 10 relevant traits. The methods with the best performance are identified with two asterisks, while the second-best PRS method is denoted with a single asterisk. The simulation settings (a)-(f) feature different genetic correlations (0.1 or 0.6) under non-overlapping (a) and (b), partially overlapping (c) and (d), and completely overlapping (e) and (f).

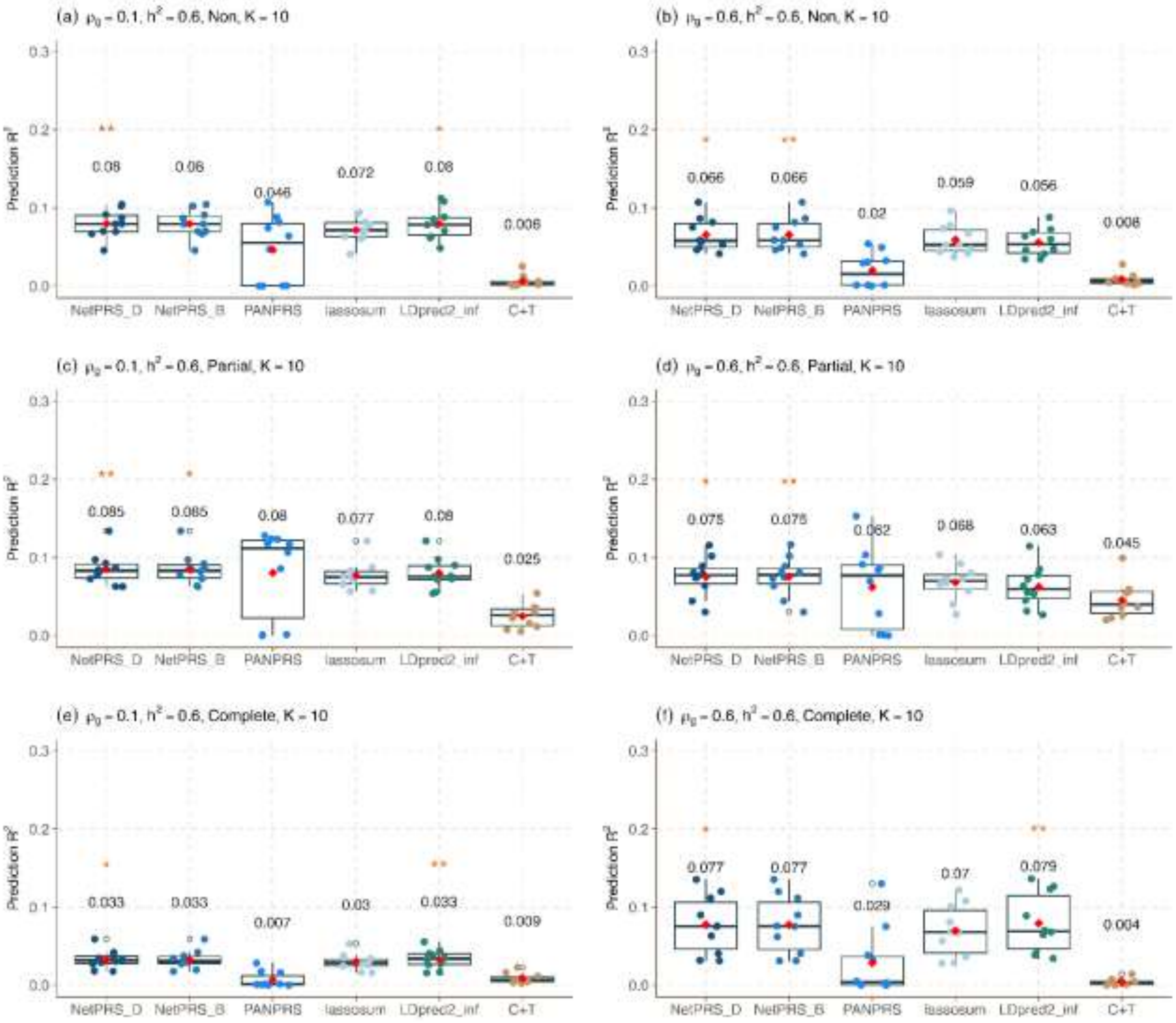

Figure S4: Predictive performances of NetPRS and other methods in simulations under different heritability scenarios. The boxplot displays the prediction R^2 across ten simulation replicates for different methods in six simulation settings. The average value of R^2 across ten replicates is shown above the boxplot. Results are presented for six scenarios with a fixed genetic correlation $\rho_g = 0.1$ and $K = 10$ correlated traits. The methods with the best performance are identified with two asterisks, while the second-best PRS method is denoted with a single asterisk. The simulation settings (a)-(f) feature different heritability (0.2 or 0.6) under non-overlapping (a) and (b), partially-overlapping (c) and (d), and completely-overlapping (e) and (f).

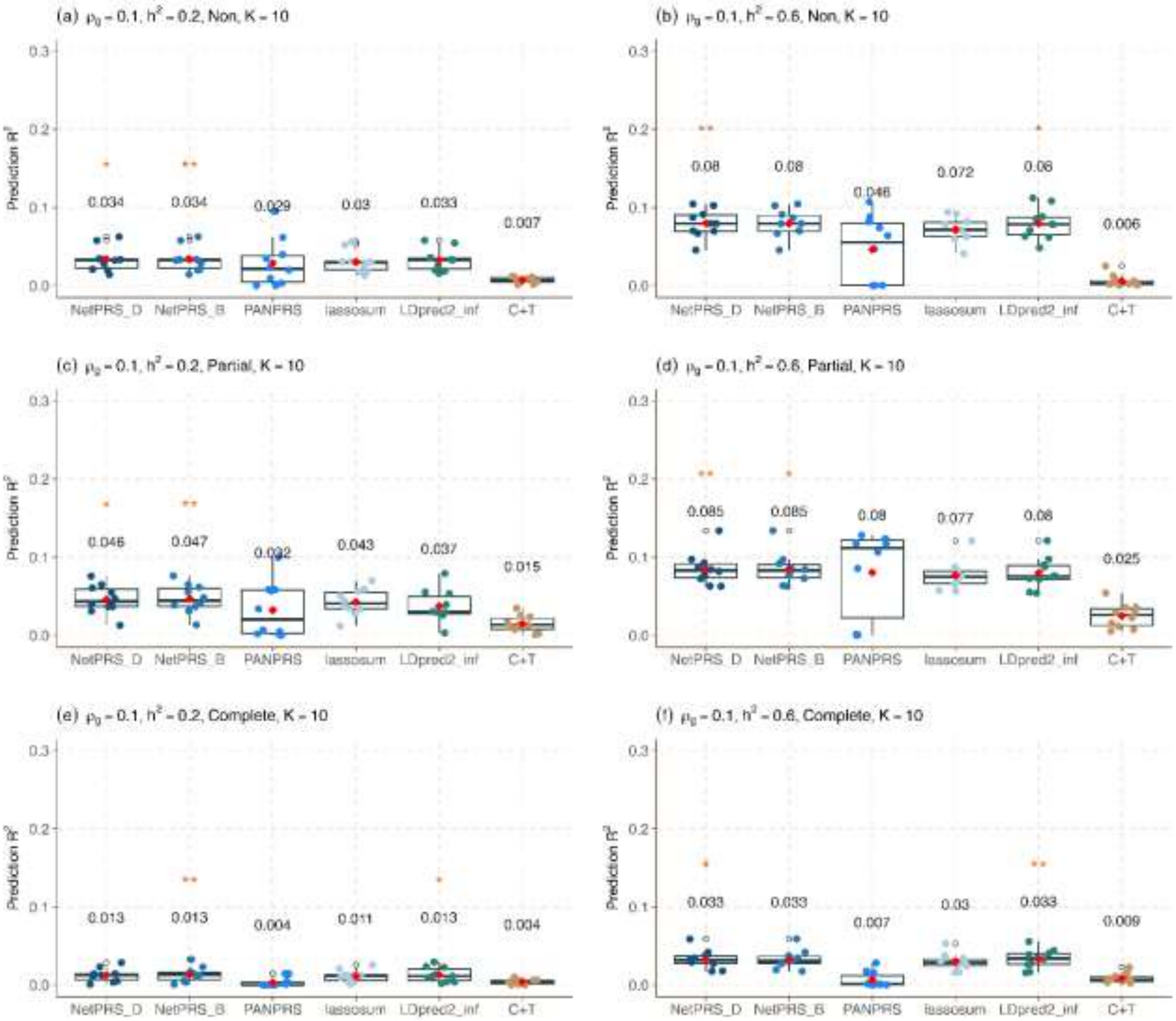

Figure S5. Predictive performances of NetPRS and other methods in simulations under different heritability scenarios. The boxplot displays the prediction R^2 across ten simulation replicates for different methods in six simulation settings. The average value of R^2 across ten replicates is shown above the boxplot. Results are presented for six scenarios with a fixed genetic correlation $\rho_g = 0.1$ and $K = 5$ correlated traits. The methods with the best performance are identified with two asterisks, while the second-best PRS method is denoted with a single asterisk. The simulation settings (a)-(f) feature different heritability (0.2 or 0.6) under non-overlapping (a) and (b), partially-overlapping (c) and (d), or completely-overlapping (e) and (f).

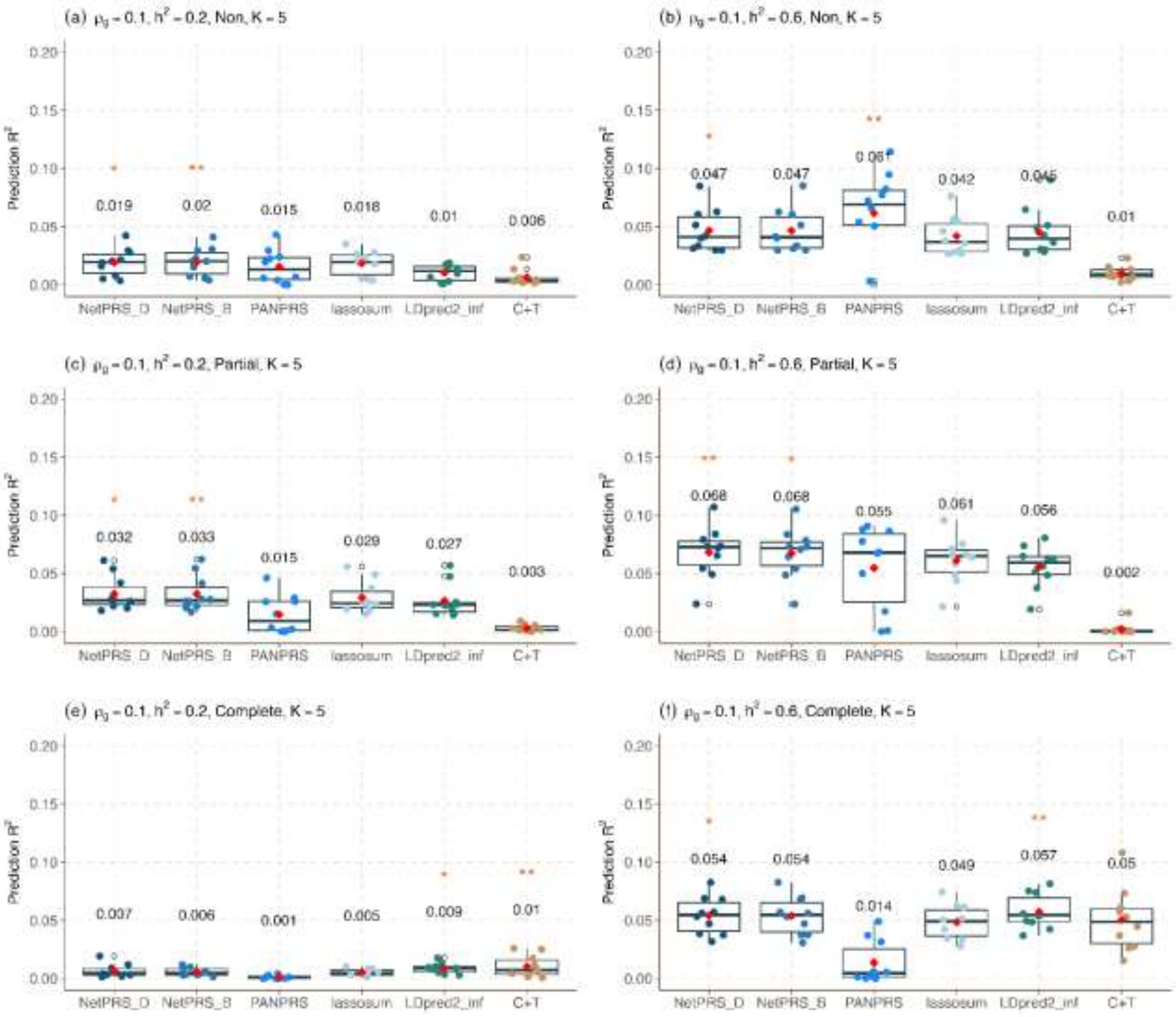

Figure S6. Predictive performances of NetPRS and other methods in simulations under different heritability scenarios. The boxplot displays the prediction R^2 across ten simulation replicates for different methods in six simulation settings. The average value of R^2 across ten replicates is shown above the boxplot. Results are presented for six scenarios with a fixed genetic correlation $\rho_g = 0.6$ and $K = 5$ correlated traits. The methods with the best performance are identified with two asterisks, while the second-best PRS method is denoted with a single asterisk. The simulation settings (a)-(f) feature different heritability (0.2 or 0.6) under non-overlapping (a) and (b), partially-overlapping (c) and (d), or completely-overlapping (e) and (f).

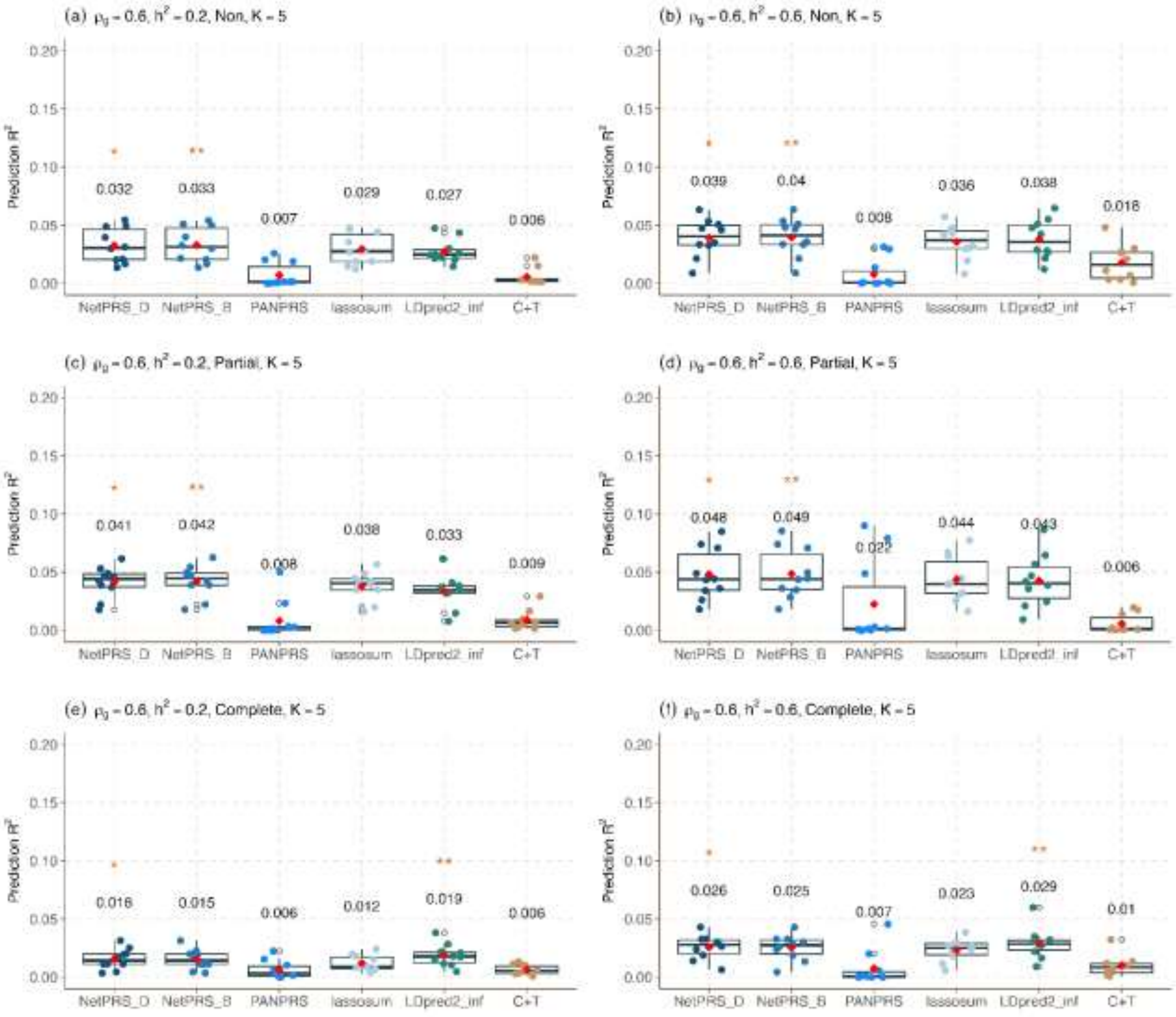

Figure S7. Predictive performances of NetPRS and other methods in simulations under different heritability scenarios. The boxplot displays the prediction R^2 across ten simulation replicates for different methods in six simulation settings. The average value of R^2 across ten replicates is shown above the boxplot. Results are presented for six scenarios with a fixed genetic correlation $\rho_g = 0.6$ and $K = 10$ correlated traits. The methods with the best performance are identified with two asterisks, while the second-best PRS method is denoted with a single asterisk. The simulation settings (a)-(f) feature different heritability (0.2 or 0.6) under non-overlapping (a) and (b), partially-overlapping (c) and (d), or completely-overlapping (e) and (f).

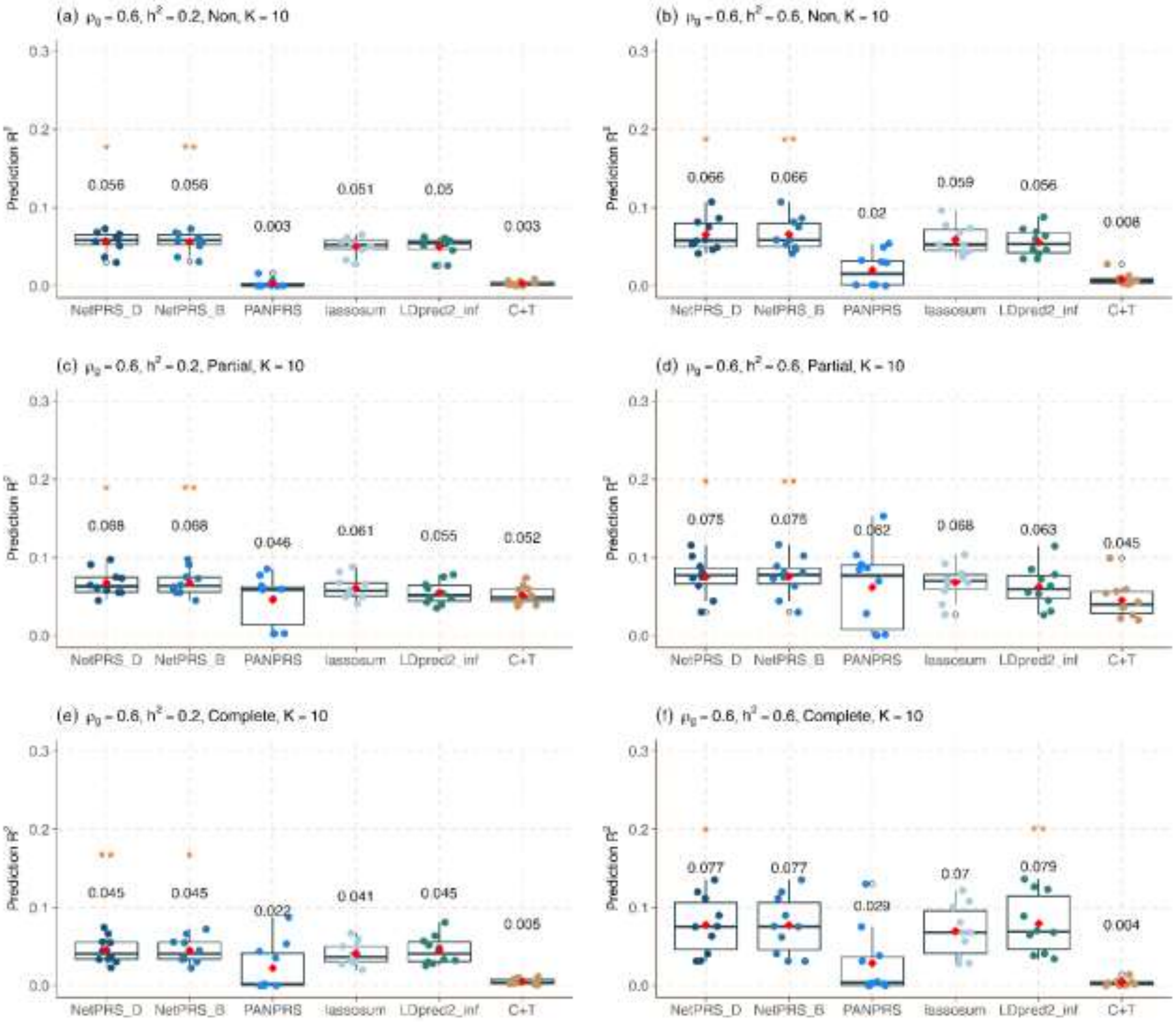

Figure S8. Predictive performances of NetPRS and other methods in simulations under different number of traits. The boxplot displays the prediction R^2 across ten simulation replicates for different methods in six simulation settings. The average value of R^2 across ten replicates is shown above the boxplot. Results are presented for six scenarios with a fixed genetic correlation $\rho_g = 0.1$ and heritability $h^2 = 0.2$. The methods with the best performance are identified with two asterisks, while the second-best PRS method is denoted with a single asterisk. The simulation settings (a)-(f) feature different number of relevant traits (5 or 10) under non-overlapping (a) and (b), partially-overlapping (c) and (d), or completely-overlapping (e) and (f).

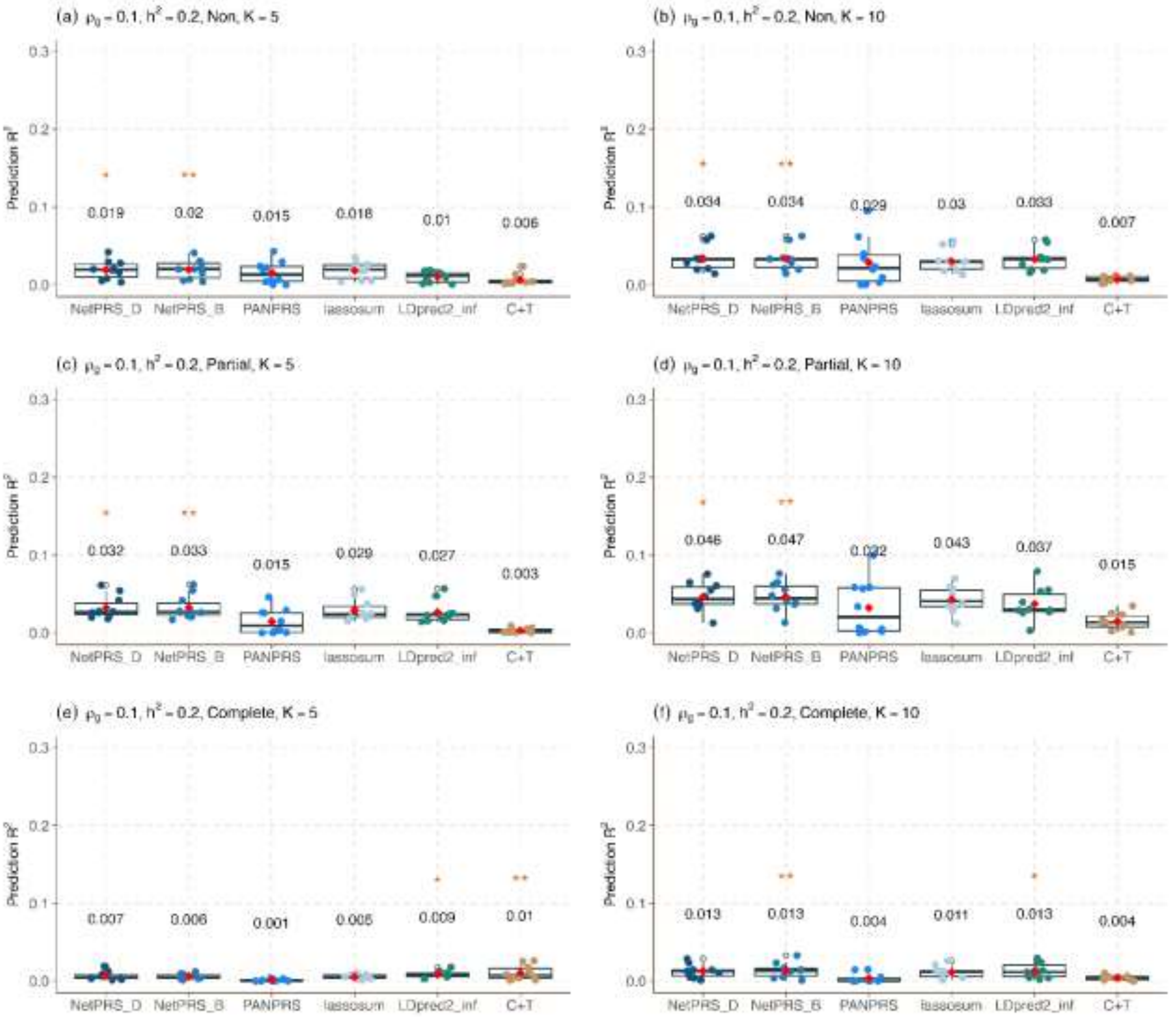

Figure S9. Comparisons of the prediction accuracy of all methods using GWAS summary statistics and individual-level data. Each dot represents the Pearson correlation of average R^2 between the individual data-based and GWAS summary-based estimation for one simulation scenario.

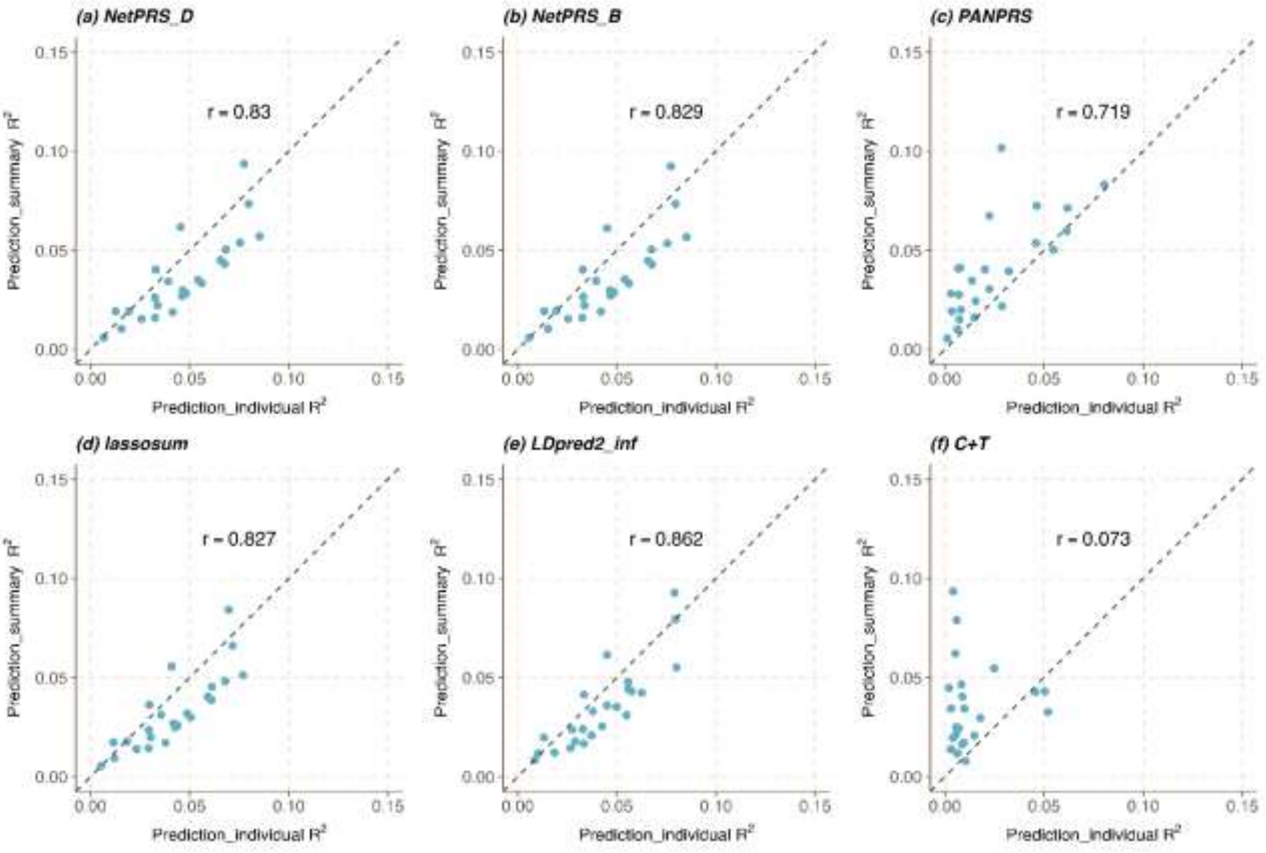

Supplement: 1 [file NIHPPRS9073777V1-supplement-1.pdf]
